# An Exploratory Study of Diabetes in a First Nation Community with Respect to Serum Concentrations of *p,p’*-DDE and PCBs and Fish Consumption

**DOI:** 10.3390/ijerph6123179

**Published:** 2009-12-11

**Authors:** Aline Philibert, Harold Schwartz, Donna Mergler

**Affiliations:** 1 CINBIOSE, Université du Québec à Montréal (UQÀM), C.P. 8888 succ. Centre ville H3C 3P8, Montréal (Québec), Canada; E-Mail: mergler.donna@uqam.ca; 2 Environmental Health Research Division, First Nations and Inuit Health Branch, Health Canada, Tunney’s Pasture, Ottawa, Ontario K1A 0K9, Canada; E-Mail: harold_schwartz@hc-sc.ca

**Keywords:** First Nation, diabetes, fish, organochlorines, DDE, PCB

## Abstract

This study examined the association between self-reported diabetes, fish consumption and serum levels of organochlorines in a First Nation community. One quarter of the 101 participants reported diabetes. Serum PCBs, but not *p,p’*-DDE, were positively correlated to consumption frequency of total fish, walleye and pike, but not trout. Reported diabetes was positively associated to *p,p’*-DDE and some PCB congeners. Odds Ratios (OR) for reported diabetes for those in the upper 75th percentile for serum *p,p’*-DDE compared to the others were 3.5 (95% CI 1–13.8) and 6.1 (95% CI 1.4–27.3) (weight wet and lipid-standardized values, respectively) and for total sum of PCBs: 4.91 (95% CI 1.4–19.0) and 5.51 (95% CI 1.3–24.1). For participants who were in the upper 50th percentile for trout and white fish intake, reported diabetes was respectively 6 and 4 times lower compared to the others. These findings support the hypothesis that environmental exposure to elevated *p,p’-*DDE and PCBs is associated with increased risk of diabetes. Consumption of trout and white fish may be beneficial to reduce risk.

## 1. Introduction

Diabetes has been one of the most important growing public health concerns for the last two decades [1–3]. At present, over 171 million people worldwide have diabetes and this number is projected to globally double by 2030 [3]. Numerous studies have attributed the diabetes epidemic to various lifestyle factors, such as obesity, lack of exercise, and poor diet [4–9]. Recent evidence suggests that exposure to some commonly encountered environmental contaminants may also contribute to Type 2 diabetes [10–11]. Several studies have shown a positive association between diabetes and biomarkers of organochlorine (OC) exposure, including several polychlorinated biphenyls (PCB) congeners and chlorinated pesticides like dichlorodiphenyldichloroethylene (*p,p’*-DDE) [12–22].

In North America, aboriginal populations present a disproportionately high prevalence of Type 2 diabetes. In Canada, the burden of Type 2 diabetes is three to five times higher in native populations than in the general Canadian population and will likely continue to increase [23–25]. High levels of OCs have been reported for northern native populations (for review see [25]), and traditional food is considered the major vector of contaminant exposure.

In 1999, the First Nations and Inuit Health Branch of Health Canada carried out an exposure assessment of environmental contaminants in the community of Kitchenuhmaykoosib Inninuwug located in the Lookout Sioux zone of Northern Ontario. The present study explored this data set with respect to a possible association between self-reported diabetes, fish consumption and serum concentrations of the OCs.

## 2. Methods

The recruitment objectives for the 1999 Health Canada Assessment was to randomly select 100 persons from the Band list of community members and to ask the Elders to select 15 persons for their high consumption of traditional food. The Band list included 525 adults (15 years old and over). Their age distribution was: 15–29 yr: 37.9%; 30–39 yr: 22.9%; 40–49 yr: 14.7%; 50–59 yr: 9.3%; 60 yr and over: 15.2%. Women comprised 52.5%. From the random selection process, a total of 200 people were randomly chosen. Of these, 79 (40%) agreed to participate in the assessment. Non-participants were either absent from the community or refused to participate. In addition, 16 people were selected by the Elders and accepted to participate; six other persons volunteered and were included in the study. A total of 101 people participated. Thus, in the final sample, compared to the underlying population, younger persons were underrepresented (<40 yr: 42.5% *vs.* 60.9%, while older persons were over-represented.

All questionnaires were administered by interview either in English or Oji-Cree. Participants were asked to provide information on socio-demographic characteristics, lifestyle, traditional food consumption (fish and wild game species) and medical history, including diabetes. Self-reported diabetes was coded as “Yes” if the participants answered having been diagnosed with diabetes. Serum samples were analyzed for eight PCB congeners and *p,p’*-DDE at the First Nations and Inuit Health Branch Laboratory of Health Canada. PCBs were analyzed by gas chromatography with electron capture detection (unpublished method). The peak areas of PCB congeners 99, 118 and 153 were used to estimate Aroclor 1254, whereas congeners 99, 153 and 180 entered into the formula for calculation of Aroclor 1260. Total sum of PCB congeners was calculated from the sum of individual congeners: 74, 99, 118, 153, 138, 187, 180 and 170. The detection limit for the quantification of Aroclor 1254 and Aroclor 1260 was 0.5 μg/L and for the PCBs congeners the limit was 0.05 μg/L. The quantification of *p,p’*-DDE was performed by gas chromatography with electron capture detector (unpublished method) using an external standard. The limit of detection and quantification was 0.05 μg/L. Samples under the detection limit were assigned half this limit.

## 3. Statistical Analysis

Because OCs are highly lipophilic compounds, serum OC values were adjusted for total serum lipid (standardized-lipid value). Multiple logistic regressions were performed with continuous variables for serum OCs and the Odds Ratio (OR) was determined, comparing the prevalence of reported diabetes for those whose OC concentrations were in the upper 75th percentile to the others. The covariables for diabetes considered here include: gender, age, place of birth, current smoking status, and total serum lipids. Similar logistic regressions were performed for the frequency of fish intake (total and specific species) controlling for the total sum of PCB congeners and the above covariables; the median was used for determining ORs. Because more than 50% of values were missing for body mass index (BMI), we were unable to consider the influence of this known risk factor for diabetes. The limit of significance was set at ≤0.05. Analyses were performed using SPSS version 16 (SPSS Inc., Chicago, IL).

## 4. Results

Participants’ age ranged from 15 to 86 years old (mean, 45 ± 17.5 yr; median, 43 yr). Women comprised half the population (51.5%) and were slightly older than men (medians 49 and 40 yr, respectively). Most (84.2%) were born in this community. More than half the men (58.4%) and one fourth of the women (27.5%) reported currently smoking. Diagnosed diabetes was reported by 25 persons (seven men and 18 women), representing 24.8% of all participants. The prevalence of self-reported diabetes in women (35.3%) was 2.5 times higher than in men (14.0%). Self-reported diabetes increased with age in both men and women (15% in those 35 years and less; 35% in those older than 35 years). The mean frequency of total fish intake was 4.64 fish meals/month (median: 2.24). Trout was the main fish consumed by the participants (mean: 2.30 fish meals/month; median 1.45 fish meals/month). Men consumed a similar number of fish meals to women, regardless of fish species.

Table 1 shows the wet weight and lipid-standardized serum values of *p,p’*-DDE and PCBs, as well as total and fish species consumption. Men and women displayed similar serum *p,p’*-DDE and PCBs concentrations except for PCB-74. Women had significant higher levels of both wet weight and lipid-standardized values of serum PCB-74 (Mann-Whitney U test, p = 0.019 and 0.02 respectively). Serum PCBs and *p,p’*-DDE were strongly and positively correlated with age, with Pearson’s rho correlation coefficients ranging from 0.79 (*p,p’*-DDE) to 0.81 (total sum of PCB congeners). Serum PCBs, but not serum DDE, were positively correlated to the frequency of total fish intake, walleye and pike (p = 0.001, 0.04 and 0.03, respectively), taking into account age and gender. No relation was observed between OCs and white fish or trout consumption.

Multiple logistic regression analyses, using continuous data for serum OCs, showed that self-reported diabetes was significantly associated with both wet weight and lipid-standardized serum *p,p’*-DDE, PCB-74, 153, Aroclor 1240 and 1260, and total sum of PCB congeners (Wald coefficients varied from 3.95 to 6.36). The Odds Ratios (OR) for self-reported diabetes for those who were in the upper 75th percentile for serum OCs compared to the others, are presented in Table 2.

All associations between self-reported diabetes and serum OCs (*p,p’*-DDE and PCBs) were consistent in gender-based stratified analyses even though some of the associations lost their statistical significance (p > 0.05). After including BMI category as a confounding variable in a reduced group (not shown), the relations between self-reported diabetes and OCs remained significant and displayed similar or higher ORs.

Analysis of self-reported diabetes with respect to total fish and fish species showed a significant inverse relation between self-reported diabetes and intake of total fish, trout and white fish (Table 3), controlling for total sum of PCB congeners and the same covariables described above. Trout intake showed the strongest relation (B coefficients: −0.28 and −0.01, for trout and white fish respectively). For participants who were in the upper 50th percentile for the frequency of trout and white fish intake, self-reported diabetes was six times (5.88) and four times lower than the others, respectively, while the relation with total fish did not reach significance. No association was found with the frequency of other fish species or game (not shown).

## 5. Discussion

The prevalence of diabetes (24.8%) among participants in the present study was higher than that reported for a community wide survey in an Oji-Cree community likewise located in Sioux Lookout Zone (17.2% [28]) in Northern Ontario, but this difference probably reflects the overrepresentation of older participants in the present survey. The median age in this study is 43 years, whereas the Aboriginal population median age is 27 years [29]. However, other geographically close First Nation communities show similar proportions ranging from 17 to 24.9% [20]. These rates of diabetes are higher than those reported for First Nation communities from the James Bay region in Ontario and Northern Quebec (from 5.2 to 9.2% [30,31]). The prevalence of diabetes in the general Canadian population varied from 4 to 5.8% between 2000 and 2005 [32,33]. Among the participants of the present study, proportionally more women than men reported diabetes, which is consistent with other First Nation studies [23–24,30–31].

The level of OC exposure for those who participated in the present study approached the range of Inuit communities who eat marine fish and mammals, and was higher than those for other First Nation communities who consume freshwater fish and terrestrial game. Indeed, mean *p,p’*-DDE of the participants from Kitchenuhmaykoosib Inninuwug was similar or approached those reported for Inuit marine fish eater communities from Baffin Island (range 0.6–6 ppb [34]) and Alaska (mean 9.02 ppb [35]) and exceeded levels reported in other First Nation communities. In Akwesasne Mohawks [16], mean *p,p’*-DDE was 537 ng/g lipids, while it varied from 412.9 to 508.9 ng/g lipids in the James Bay communities [36]. In the present study, PCB-74 was similar to Inuit communities of Nunavik (60 ng/g [37]), but PCB-153 was lower than in Nunavik (1,270 ng/g lipids and 3.17 μg/L respectively [37,38]). Both these congeners were higher than the levels reported for First Nation communities in James Bay (PCB-153: 90 to 134 ng/g lipids [36]) and for Akwesasne Mohawks (PCB-74: 0.25 to 0.33 μg/L; [20,39,40]. A1260 showed higher levels comparatively to that found in First Nation communities in James Bay (between 730 and 1146 ng/g lipids, [36]. The high concentrations of *p,p’*-DDE, PCB-74 and PCB-153 may reflect selection bias, but may also reflect past point sources of exposure within this community.

The associations between self-reported diabetes, serum *p,p’*-DDE, PCB-74 and PCB-153 are consistent with the findings of Codru *et al*. [16], who carried out a study in aboriginal communities, and with those of other studies with non-native populations [14,15,17,18]. Although the cross-sectional nature of our study does not allow us to establish a causal relationship, these findings, coupled to previous studies, suggest a higher risk of diabetes in relation to OC exposure (*p,p’*-DDE and PCBs). However, a reversed causality cannot be excluded because disease induced metabolic perturbations may facilitate the accumulation and/or affect metabolism of OCs [14].

The Kitchenuhmaykoosib Inninuwug First Nation community is located at 53° 49’1” N; 89°52’30” W, on the North Shore of Big Trout Lake, the largest lake in Northwestern, Ontario, Canada: 58 km. long and 29 km. wide. It is a “fly in” community and can only be reached by road in winter over the frozen lake. Fish is an important part of the diet. There was a positive association between consumption of several fish species and serum PCBs, but not for trout or whitefish, probably reflecting inter-species differences in PCB bioaccumulation [41–43]. No relation was observed between fish consumption and *p,p’-*DDE.

In this study, the positive effects of fish consumption on diabetes were observed particularly for trout and whitefish. Differences between fish species probably reflect their nutrient content. For example, trout and whitefish are known to have high levels of DHA, while walleye and pike have much lower low levels [41]. There may also be differences in absorption of omega-3 fatty acids from fatty and lean fish [44]. The protective effects of certain fish species on the risk of diabetes support the potential benefits of traditional foods to improve health status of First Nation communities [23]. Future studies of Type 2 diabetes in First Nation communities need to consider specific fish species consumption, serum omega-3 fatty acids and other nutrients, as well as OC exposure.

There are several limitations to this exploratory study, which analyzed data collected in a larger project, undertaken to primarily assess the exposure levels of environmental contaminants in this population. Only 40% of those selected from the Band lists participated in the study and younger persons were underrepresented. Several factors may explain this low participation rate, including absence from the community, illness, lack of interest and/or reticence to participate in government organized events. Diabetes was not diagnosed but self-reported, and Type 1 and 2 diabetes were not differentiated. However, since all participants reported the onset of diabetes after 20 yrs old, it is probably mostly type 2 diabetes. Although the participants were asked to fast overnight before blood sampling interview, we cannot confirm they actually did. However there is no reason to think the participants with the higher serum levels in *p,p’*-DDE and PCBs systematically did not fast. Although the missing values of BMI for most participants prevented us from considering the influence of this known risk factor of diabetes in the study population, the similar results found in a reduced group support the present findings.

The findings of this exploratory study underline the importance of considering fish species both with respect to risk and benefits. However, the negative association of diabetes with the frequency of trout consumption demonstrates that certain traditional foods may protect First Nations from diabetes. Large-scale studies of diabetes and other chronic diseases among First Nation populations should examine the possible opposing contribution of contaminants and specific species of fish and game in order to maximize the benefits from traditional food and minimize the risk from exposure to toxic substances.

## Figures and Tables

**Table 1.** Characteristics of mean (min-max) and percentiles of wet weight and lipid-standardized values of serum *p,p’*-DDE and PCBs and frequency of fish intake.

**Variables**	**% Detected**	**Mean**	**Min**	**Max**	**Percentiles**		
					**25**	**50**	**75**
*Organochlorines*							
*Weight wet values (ppb)*							
*p,p’*-DDE	100	7.52	0.13	87.15	0.92	3.06	10.65
PCBs :							
PCB-74	66.3	0.35	0.03	2.60	0.03	0.12	0.55
PCB-153	99.0	2.54	0.03	13.20	0.40	1.27	3.25
A1254	94.1	19.75	0.25	125.40	1.95	6.50	17.40
A1260	94.1	22.38	0.25	134.40	2.25	8.50	21.85
Total sum of PCB congeners		8.24	0.20	42.00	1.21	3.97	10.58
*Lipid-standardized values (ng/g lipid)*							
*p,p’*-DDE		1293	23.30	19431	175.38	536.51	1617
PCBs :							
PCB-74		58.41	3.74	579.71	5.60	20.03	83.30
PCB-153		28.17	5.54	2 620	72.23	234.75	607.97
A1254		3289	44.81	24811	361.37	1220	3707
A1260		3728	47.60	26153	432.62	1604	4827
Total sum of PCB congeners		1384	44.33	8863	219.94	742.56	1 946
*Frequency of fish intake (fish/month)*							
Total fish		4.64	0	25	0.83	2.24	6.00
Trout		2.30	0	20	0.33	1.45	2.75
Walley		1.05	0	5	0	0.33	1.45
White fish		0.76	0	15	0	0	0.45
Pike		0.39	0	5	0	0	0.25

Mean values and percentiles were calculated with non detectable values transformed to 0. Abbrevations: LOD, level of detection; A1254, Aroclor 1254; A1260; Aroclor 1260. Total sum of PCB congeners (sum of individual congeners), PCB-74, 99, 118, 138, 153, 170, 180 and 187. Total fish frequency is the sum of all fish species consumed per month.

**Table 2.** Association between self-reported diabetes (Yes or No), wet weight and lipid-standardized values serum of *p,p’*-DDE, individual PCB congeners PCB-74 and PCB-153, Aroclor 1254 and 1260, and total sum of PCB congeners.

**Organochlorines**	**Wet weight values (μg/L)**			**Lipid-standardized values (ng/g)**		
	Odds ratio	95% CI	p	Odds ratio	95% CI	p
*≤ or > 75th percentiles*						
*p,p’*-DDE	6.11	1.37–27.30	0.018	3.56	0.97–13.08	0.050
*PCBs*						
PCB-74	4.39	1.15–16.83	0.030	6.06	1.21–30.27	0.028
PCB-153	4.91	1.27–19.01	0.021	6.46	2.07–36.63	0.020
A1254	4.89	1.05–18.59	0.043	4.28	1.02–18.03	0.046
A1260	4.91	1.27–19.01	0.021	4.52	1.10–18.54	0.036
Total sum of PCB congeners	4.91	1.27–19.01	0.021	5.51	1.26–24.07	0.023

Abbrevations: A1254, Aroclor 1254; A1260; Aroclor 1260. Total sum of PCB congeners (sum of individual congeners), PCB-74, 99, 118, 138, 153, 170, 180 and 187. The OR were controlled for gender, age (≤ or > 45 yr), place of birth (Kitchenuhmaykoosib Inninuwug Yes or No), current smoking status (Yes or No) and total lipids (mg/dL) for non lipid-standardized values.

**Table 3.** Association between self-reported diabetes (Yes or No) and the frequency of fish intake (fish/month).

Fish consumption	Odd ratios	IC 95%	P value
Continuous variable *(fish/month)*			
Total fish	0.98	0.97–0.99	0.04
Trout	0.75	0.58–0.98	0.03
Walleye	0.90	0.27–1.00	0.61
White fish	0.48	0.22–1.05	0.07
Pike	0.62	0.31–1.23	0.73
<= or > 50% percentile			
Total fish	0.16	0.03–1.02	0.06
Trout	0.17	0.03–0.97	0.03
Walleye	1.31	0.37–4.74	0.68
White fish	0.25	0.06–0.95	0.04
Pike	0.33	0.08–1.45	0.14

The OR were controlled for gender, age (≤ or > 45 yrs), Place of birth (Kitchenuhmaykoosib Inninuwug Yes or No), current smoking status (Yes or No) and total sum of PCB congeners (sum of individual congeners), PCB-74, 99, 118, 138, 153, 170, 180 and 187.
